# Clinicopathological Analysis of Five Cases of NUT Midline Carcinoma, including One with the Gingiva

**DOI:** 10.1155/2020/9791208

**Published:** 2020-02-13

**Authors:** Luting Zhou, Xiang Yong, Jun Zhou, Jiankun Xu, Chaofu Wang

**Affiliations:** ^1^Department of Pathology, Ruijin Hospital, Shanghai Jiaotong University School of Medicine, Shanghai 200025, China; ^2^Department of Pathology, The Third Affiliated Hospital of Bengbu Medical College, Suzhou 234000, China

## Abstract

**Aims:**

NUT midline carcinoma (NMC) is a rare, poorly differentiated carcinoma defined by the presence of NUT gene rearrangement. In order to better understand the diagnostic and clinicopathologic features of this disease as they pertain to clinical practice, we have herein compiled findings pertaining to 5 cases of NMC at our institution.

**Methods:**

Clinicopathological findings from 5 NMC cases were retrospectively reviewed, with histologic findings being reassessed and summarized accordingly. Tumor samples in the present study had been stained for markers including NUT, P63, P40, TTF-1, keratin, CK7, Syn, CD56, CgA, CD34, CD117, EGFR, and Ki-67. All cases were subjected to both fluorescence in situ hybridization (FISH) and followed up.

**Results:**

Of these 5 NMC cases, 2 were males and 3 were females, with ages ranging from 26 to 69 years. A total of 2 cases localized to the lung, 1 to the larynx, 1 to the maxillary gingiva, and 1 to the orbital cavity. Upon microscopic assessment, these tumors appeared as clusters of small rounded cells with interstitial neutrophil infiltration. Squamous epithelial differentiation varied between samples. NUT staining revealed strong diffuse nuclear staining in tumor cells, and FISH confirmed the presence of NUT gene translocation in these samples.

**Conclusions:**

NMC is a form of highly invasive cancer that can manifest in a number of tissues including the gingiva. NMC tumors have a fairly well-defined pathological morphology, and both immunohistochemistry and FISH are valuable for NMC diagnosis.

## 1. Introduction

NUT midline carcinoma (NMC), also known as t(15; 19) carcinoma, is a form of rare, aggressive, poorly differentiated cancer that is defined by the presence of a gene rearrangement in the NUT gene, which encodes a nuclear protein normally expressed in the testes. To date, under 1000 total NMC cases have been reported, with the first report coming from Kubonishi et al. in 1991 [1], who detected a thymic carcinoma with a novel t(15; 19) (q15; p13) chromosomal abnormality. Roughly a decade later, French et al. helped to define NMC as a distinct clinicopathological entity [2, 3]. While NMC tumors most often arise in the midline regions in or near the upper airway such as the head, neck, and thorax, they have also been detected in sights including the lungs, salivary glands, pancreas, bladder, kidneys, soft tissues, and bones [4–7]. No previous reports have described NMC tumors arising in the gingiva. Rates of NMC in males and females have been found to be roughly equal, and while originally identified in children and young adults, the more recent work suggests that NMC can affect individuals of all ages. NMC is extremely aggressive, with a median survival of 7 months, and no specific chemotherapy regimen has demonstrated efficacy in treating NMC [8].

Approximately 70% of NMC cases present with a translocation of the BRD4 gene, resulting in a roughly 6.4 kb BRD4-NUT fusion gene. In the remaining 30% of NMC cases, NUT fuses with BRD3 or other unknown genes [9, 10].

In many instances, NMC can be misdiagnosed as another form of poorly differentiated tumor. The only definitive way to diagnose NMC is through the detection of NUT gene translocation via either immunohistochemistry, fluorescence in situ hybridization (FISH), or gene sequencing.

Herein, we report on 5 cases of NMC who presented to our institution, including one case arising in the maxillary gingiva, which has never been described previously. We summarize both the clinical and pathological features of NMC and discuss the differential diagnosis for these tumors in order to better understand this rare and aggressive disease.

## 2. Materials and Methods

### 2.1. Patients and Case Selection

This study was approved by the Institutional Review Board of the Department of Pathology, Ruijin Hospital, Shanghai Jiaotong University School of Medicine. In total, 5 cases of NMC were retrospectively analyzed as a part of this study. For each of these cases, complete clinical data were reviewed, and all available hematoxylin and eosin- (H&E-) stained slides were analyzed independently by two experienced pathologists (LT. Z. and X. Y.). In addition, patient follow-up was conducted via telephone interview.

### 2.2. Immunohistochemistry

Available tissue sections were used for immunohistochemistry (IHC) analyses of these 5 NMC cases. Surgical tissue sections were sectioned at 4 *μ*m thickness and then stained with primary antibodies specific for NUT (ab122649, 1 : 500, Abcam, UK), CK (AE1/AE3, prediluted; Dako, Denmark), vimentin (V9, prediluted; Dako), CK7 (EP16, 1 : 200, ZSGB‐BIO, China), SMA (1A4, 1 : 200, ZSGB-BIO), CD34 (QBEND, prediluted, Dako), p63 (ZM-0406, prediluted, ZSGB‐BIO), p40 (ZA-0483, 1 : 100, ZSGB‐BIO), Syn (DAK-SYNAP, prediluted, Dako), CD56 (123C3, prediluted, Dako), CgA (DAK-A3, prediluted, Dako), and Ki-67 (MIB-1, prediluted, Dako). A Dako Omnis automated staining platform was used for the IHC staining of these formalin-fixed paraffin-embedded whole tissue sections. Appropriate positive controls were used for all assays.

### 2.3. Fluorescence In Situ Hybridization

Translocations of the NUTM1 gene at 15q14 were analyzed via FISH using the ZytoLight SPEC NUTM1 Dual Color Break Apart Probe. Samples were first fixed for 24 h with 10% neutrally buffered formalin at room temperature, embedded at a temperature below 65°C, and sliced into 2–4 *μ*m thick sections via microtome. Sample pretreatment was conducted based on the instructions provided with the ZytoLight FISH-Tissue Implementation Kit, and an Olympus BX51TRF microscope (Olympus, Japan) was used for FISH signal analysis with a triple-pass filter (DAPI/Green/Orange; Vysis). Samples were deemed to have a positive FISH signal when there was a distance between red and green signals of ≥2 signal diameters.

## 3. Results and Discussion

### 3.1. Clinical Features

**Figure 1:**
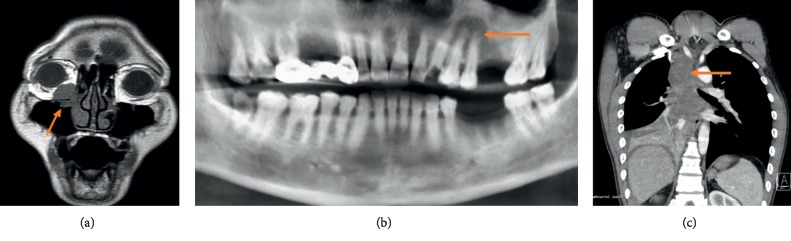
NUT carcinoma imaging characteristics. MRI scans revealing a 1 cm mass infiltrating the right orbit (a). CT scans revealing the presence of a 2 cm × 1.5 cm ovoid shadow at the apex of teeth 11-12 surrounded by dense white lines and an unclear boundary (b). CT scans indicating the presence of an area of soft tissue of irregular density within the right lung hilum, with contrast-enhanced images revealing the presence of nonhomogeneous enhancement and local necrosis (c).

Key clinical findings for patients in this study are compiled in Table 1. Of these 5 cases, 3 were females and 2 were males, with ages from 26–69 years. Case 1 was a 59-year-old female who originally presented with complaints of an abnormal sensation in the right orbit for the last ∼15 days. Examination of this patient revealed a 1 cm × 1 cm × 1 cm mass that was visible within the right orbit. This mass was adherent to surrounding tissues and had poor mobility. A magnetic resonance imaging (MRI) scan confirmed the presence of a 1 cm infiltrative mass within the right orbit (Figure 1(a)). Case 2 was a 35-year-old female who presented with complaints of dyspnea and dysphagia over the preceding 2 months and was diagnosed with an NMC tumor of the larynx. Case 3 was a 38-year-old female who presented with a mass in the left maxillary gingiva that had been present for 2 months. A computed tomography (CT) assessment of this patient revealed a 2 cm × 1.5 cm transparent ovoid shadow at the apex of teeth 11-12. This mass was surrounded by dense white lines and had a poorly defined boundary (Figure 1(b)). These patients had no family history of inherited diseases.

The remaining 2 cases of NMC in the present study were found in the lungs of affected patients. Case 4 was a 26-year-old male who presented with fever, shortness of breath, and a dry cough that had been present for 5 days. The symptoms of this patient had been progressing rapidly, and he exhibited pronounced shortness of breath and difficulty lying on his back. A tube endoscopy of this patient revealed the presence of enlarged protuberances, nodular hyperplasia, and mucosal protuberance at the anterior wall of the lower trachea, together with obstruction of the right middle bronchial lumen. CT scans revealed atelectasis of the middle and lower lobes of the right lung, with dense patchy shadows that were evenly enhanced following enhancement, together with mediastinal and right lower hilar lymphadenopathy. Case 5 was a 69-year-old male that had presented with chest tightness and asthma over the preceding 10 days. CT scans revealed the presence of soft tissue masses with poorly defined margins in the right hilum of the lung (Figure 1(c)). This patient also presented with visible lymph node metastasis.

### 3.2. Pathologic Features

**Figure 2:**
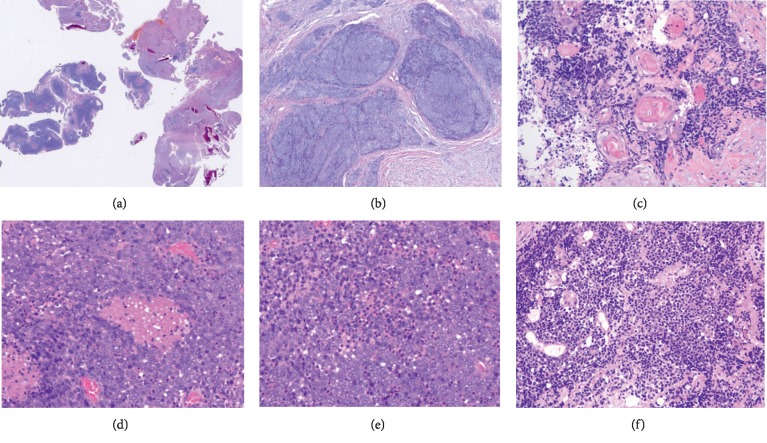
NUT carcinoma histological features. NUT carcinomas of the gingiva exhibit fragmented tissue and tumor cell clusters, with locally visible bone fragments present within the gingiva (10x) (a). Poorly differentiated tumor cells with large nuclei are evident in dense clusters separated by fibrous stroma in another region of the gingival NMC tumor (40x) (b). Abrupt regions of focal keratinization are evident in the case of orbital NMC (100x) (c). Focal necrosis is evident in the case of laryngeal NMC (d). Neutrophil infiltration is prominent in the case or laryngeal NMC (200x) (e). Focal squamous differentiation is evident in the case of orbital NMC (100x) (f).

For these cases, hemorrhage and necrosis were frequently detected within tumors. From a morphological perspective, these tumors were composed of poorly differentiated cells arranged in clusters that were separated by fibrous stroma. These tumors commonly exhibited poor adhesion and large areas of coagulative necrosis. Cells had relatively substantial nuclear and cytoplasmic contents, with uniform nuclei, numerous mitotic figures, and evident nucleoli. All 5 cases exhibited interstitial neutrophil infiltration. Keratosis was evident in cases 1 and 2, but not in case 3, while in cases 4 and 5, it was present in the gingiva and lung (Figure 2).

**Figure 3:**
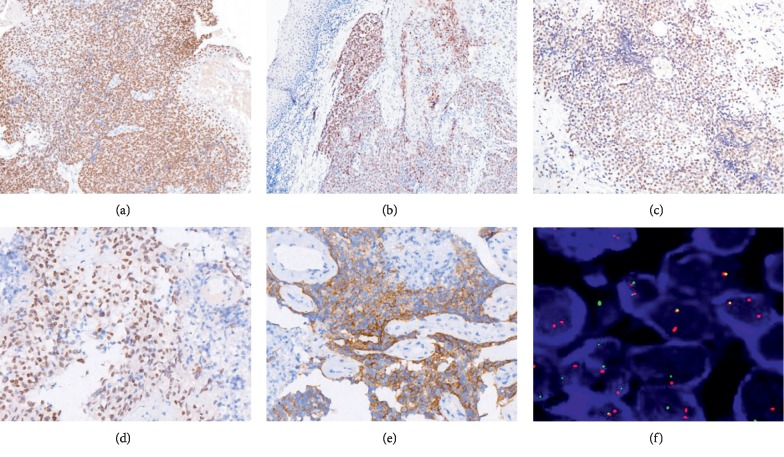
NUT carcinoma immunohistochemical and molecular features. Diffuse nuclear NUT IHC staining for the case of gingival NUT carcinoma (100x) (a), laryngeal NUT carcinoma (100x) (b), orbital NUT carcinoma (100x) (c), and lung NUT carcinoma (100x) (d). Diffuse nuclear EGFR IHC staining for the case of lung NUT carcinoma (100x) (e). NUT break-apart probe FISH revealed the presence of distinct red and green signals consistent with NUT gene translocations in all 5 cases (1000x) (f).

IHC staining of these tumor samples confirmed all cells to be positive for NUT (Figures 3(a)–3(d)) and for CK. A total of three cases were positive for P63 and P40, while all five cases were negative for TTF-1, Syn, CD56, CgA, CD34, and CD117 expression. Both cases of lung NMC were AFP negative. One case of lung NMC stained positive for EGFR (Figure 3(e)).

FISH was conducted using a NUT-specific probe to identify NUT gene locus rearrangements with an appropriate probe such that distinct red and green signals corresponded to translocations within this region. All 5 NMC cases in the present study presented with dual color FISH probe staining consistent with the translocation of the NUT gene locus (Figure 3(f)).

### 3.3. Patient Treatment and Follow-Up

NMC has a poor prognosis. As reflected in Table 1, the two patients with cases of lung NMC died within 5-6 months of diagnosis. In both cases, tumors were initially misdiagnosed as small-cell carcinomas and were subjected to etoposide chemotherapy with poor efficacy. The patient with NMC of the larynx died 9 months postdiagnosis after receiving cisplatin chemotherapy. The patient with orbital NMC died for 4 months postdiagnosis without treatment. The patient with NMC of the gingiva was alive at 6 months postsurgical resection of the tumor.

## 4. Discussion

NMC is a form of rare, aggressive, and poorly differentiated disease that can affect many organs but that most often originates in the midline regions of the head and neck, lung, and mediastinum, although it has also been reported in other tissues including the kidney, bladder, liver, salivary glands, and pancreas [11, 12]. Herein, we have reported on a series of 5 cases of NMC, including one case of a NUT carcinoma arising in the gingiva, which has never previously been reported. We summarized the clinicopathological features of this case of gingival NMC and further discussed the findings for 4 other cases affecting the lungs, larynx, and orbit. NMC can affect males and females with equal frequency, arising in individuals of all ages although cases are most common in teenagers and young adults. In the 5 cases reported in the present study, the median patient age was 45 years. NMC tumors are highly invasive, with most patients exhibiting metastasis upon diagnosis. The lymph nodes, bones, and lungs are the most common sites of NMC metastasis. In one case of lung NMC reported herein, lymph node metastasis was evident upon initial patient presentation.

The exact etiology of NMC remains to be fully clarified. Previous studies have found it to be unrelated to Epstein–Barr virus or hepatitis B virus infections. NMC patients have also not been found to exhibit a history of smoking. Somatic gene rearrangements of the NUT gene locus appear to be the basis for the development of NMC, with a single clonal translocation of the MUT (NUTM1) gene in the q14 region of chromosome 15 being evident in 100% of NMC cases. In roughly 70% of these cases, the NUTM1 gene exhibits a reciprocal translocation with BRD4 on chromosome 19p13.1 [13, 14], resulting in the formation of a BRD4-NUT fusion gene. In other cases, NUTM1 has been found to be fused with BRD3, NSD3, ZNF532, ZNF592, CIC, BCOR1, MYXD1, MYXD4, or MGA [15–18].

Based on morphological findings alone, NMC diagnosis can be extremely challenging as microscopic findings are largely nonspecific and can range from a completely undifferentiated carcinoma to a carcinoma with squamous differentiation and abrupt keratinization that may be either focal or prominent. NMC tumor cells are often epithelioid in appearance, with a rounded or ovoid nucleus, clearly visible nucleoli, high rates of mitosis, and clear evidence of necrosis and neutrophil infiltration. NUT expression was evident in all tumors upon IHC staining, with speckled nuclear positivity in >50% of tumor cells using a highly specific monoclonal nuclear NUT antibody [19]. The staining patterns for other epithelial markers including cytokeratins and carcinoembryonic antigen (CEA) were more variable. Nuclear p63 and p40 staining were evident in most cases, and CD34 positivity is helpful for distinguishing between NMC cases and leukemia. NMC tumors are also always negative for chromogranin and synaptophysin staining. EGFR positive expression in NMC tumors is very rare, with Maffini et al. having reported on one case of NMC with a EWSR1 rearrangement and strong EGFR expression [20].

In the present study, strong NUT staining was evident in all 5 cases, whereas all 5 were negative for CD34, chromogranin, and synaptophysin, and only 1 was positive for EGFR. NUT translocation was detectable by FSH in all cases. A combination of IHC and FISH analysis allows for NUT diagnosis with sensitivity and specificity values approaching 100%.

NMC has an extensive differential diagnosis including squamous cell carcinoma, small-cell carcinoma, basal cell carcinoma, Ewing sarcoma, metastatic germ cell tumor, mucosal melanoma, lymphoma, and rhabdomyosarcoma. NUT carcinoma is frequently misdiagnosed as poorly differentiated squamous cell carcinoma, as NMC tumors often exhibit local squamous differentiation and squamous cell carcinoma marker expression. However, squamous cell carcinoma is NUT-negative upon IHC analysis. NMC has a significantly poorer prognosis than does squamous cell carcinoma, making accurate diagnosis essential in order to accurately predict patient outcomes.

It is important that NMC be differentiated from small-cell carcinomas. Small-cell carcinomas exhibit a histological morphology similar to that of midline carcinomas, but without abrupt focal keratinization. These tumors also typically stain positive for neuroendocrine markers, CD56, and TTF-1, whereas they stain negative for NUT. Basal cell carcinomas may exhibit squamous epithelial differentiation and focal keratinization, but these tumors most often arise in superficial areas closely associated with the epidermis. Cells surrounding these tumor clusters are typically arranged in a palisade formation, with clear melanin deposition within tumor cells. Ewing sarcoma tumors do not exhibit squamous differentiation, with diffuse positive CD99 IHC staining and negative CK and NUT staining. FISH can confirm the presence of an EWS-FLI-1 fusion gene in these tumors.

NMC is a very aggressive disease, with patients having a median survival of only 7 months [8]. Survival rates are not associated with patient age, sex, or translocation type. To date, no chemotherapeutic regimen has been found to effectively control NMC, although studies of NMC treatment using bromodomain inhibitors are ongoing. Of the 5 NMC cases in the present study, 4 patients died within 1 year of diagnosis, while the patient with NMC of the gingiva was still alive as of the most recent follow-up.

## 5. Conclusions

In summary, NMC is a form of rare yet highly aggressive and invasive cancer. Morphological features of NMC include focal keratinization and poorly differentiated clusters of tumor cells. In addition to better-documented sites, NMC tumors can arise in the orbit and the gingiva. NUT gene rearrangement was evident in all NMC cases, as detected by FISH. As additional cases of NMC continue to be identified and studied, further insight into the cellular and molecular origins of these tumors will be gained, thereby helping to identify viable therapeutic strategies and to improve patient prognosis.

## Figures and Tables

**Table 1:** Clinicopathological characteristics of NUT carcinomas.

Case	Gender	Age (years)	Location	Size (cm)	Treatment	Died of disease
1	Female	59	Orbit	1 × 1 × 1	Without treatment	4 months
2	Female	35	Larynx	3 × 2 × 1	Cisplatin chemotherapy	9 months
3	Female	38	Gingiva	2 × 1.5 × 1	Surgical resection	AWD
4	Male	26	Lung	2 × 2 × 2	Etoposide chemotherapy	5 months
5	Male	69	Lung	5 × 4.8	Etoposide chemotherapy	6 months

AWD: alive with diseases.

## Data Availability

The data used to support the findings of this study are available from the corresponding author upon request.
